# Supplementary material for: When does risk outweigh reward? Identifying potential scoring strategies with netball’s new two-point rule
Source: PLoS One. 2020 Nov 23;15(11):e0242716. doi: 10.1371/journal.pone.0242716 (PMC7682905; doi:10.1371/journal.pone.0242716)
Supplement: Attachment — Submitted filename: Response.docx [file pone.0242716.s001.docx]

**AUTHORS RESPONSE TO REVIEWER COMMENTS**

Comments from reviewers have been presented in **bold** **text**; and comments from reviewers #1 and #2, and editorial comments have been listed as C1.X, C2.X and E.X, respectively, with ascending numbers used to label these comments. Responses to comments are in plain text and have been labelled using a similar format (e.g. R1.1 indicates the response to comment C1.1). Where revisions have been made they are referred to using line numbers of the revised manuscript, and these changes have been highlighted in the revised manuscript using **bold red text**.

**Reviewer #1**

**C1.1: Overall, the topic is interesting and worthy of scientific attention. From a scientific perspective the data analysis was performed well. My major concern is that the 2018 SSN data analysed was from prior to the two-point rule being implemented. The motivation and tactical strategy for players to shoot from outer edge (3 m to 4.9 m) of the circle was not present in 2018. The two-point rule would presumably change the tactics and subsequent behaviours of players. As such, the results are not representative of the current rule change. Furthermore, the Fast5 is not representative of either the old or new rules, given the 3.5 m threshold, less assisting players and faster pace. While these are acknowledged as limitations, I think the data does not truly support the Discussion.**

R1.1: We’d like to thank reviewer #1 for their insightful and useful comments that have hopefully improved the manuscript in this round of reviews. We agree that there are certain limitations with our approach of using existing data – and as reviewer #1 has pointed out, we attempt to appropriately acknowledge these in the limitations section of the manuscript. We hope that our responses to specific comments help address these general concerns, and have also provided further response to this in comment/response 1.6 below.

**C1.2: How does the 12.05% of shots from greater than 3m compare to now, when the two-point rule is in place?**

R1.2: While it is tempting now that the 2020 season is underway to take a glimpse at these data, our original proposed aims/research questions were focused on existing data to potentially inform team strategy around the new two-point rule. As such, we have deemed it appropriate to retain this focus – and that analysis of 2020 data will form the basis of future studies of which we can compare our original analysis from the present study to. As alluded to with this comment – it is likely that ‘long-range’ shot frequency (or the proportion of shots taken from this distance) has increased with the two-point rule in place. We have included specific reference to the potential for comparing the rate of shots > 3.0m presented in our study to future work from the 2020 (and potential future) Super Netball seasons in the revised submission (see lines 328-332 in revised submission).

*“It is quite plausible that teams will dramatically increase their proportion of shots from greater than 3.0m when the two-point shot is on offer. The 12.05% rate from a past season presented in our study provides a baseline comparator for future work examining the 2020 (and potential future) Super Netball seasons.”*

**C1.3: Were the shots from 3 m to 4.9 m in the 2018 SSN season made because of time pressures, defensive structures, or were they genuine attempts at goal as they would be with the new scoring rule? It is difficult to know.**

R1.3: As noted by reviewer #1 here, the answer to this question is quite difficult to know – however, it is likely a combination of the various factors pointed out here (i.e. some attempts due to time pressures, others due to defensive structures allowing these shots etc.). It’s likely that the reasons here will differ to those with the new rule in place (i.e. specific focused attempts at long-range shots). We have added a point on this in the limitations of the revised submission as a consideration when comparing our findings to data from when the rule is in place (see lines 436-442 in revised submission).

*“Similarly, the reasoning for players taking ‘long-range’ shots (e.g. time constraints, reacting to opposition defensive strategies vs. specifically positioning for long range shots) is likely to change with the two-point rule in place. This is another likely reason for the differences we observed between Super Netball and Fast5 data, and will be an important consideration in assessing data from the 2020 Super Netball season and beyond.”*

**C1.4: I think some practical considerations should be discussed to highlight how the new rule may influence the way SSN is played. For example, how have tactics changed and how will this influence team selection (i.e. a good close shooter and a good long-range shooter). This will no doubt influence how teams now recruit players as well.**

R1.4: Thank you for raising this – and it is an extremely valid point from a practical/coaching perspective. We believe the main elements to consider from our analysis relate to the combination of players in shooting positions and the attacking strategies required to generate shot opportunities relative to different distances to goal. Similarly, defensive strategies and line-ups likely need to adapt in response to their opponents choices. We have included extra discussion on these practical considerations in the revised submission (see lines 305-320 in revised submission).

*“Our analysis also provides some important tactical considerations around a team’s line-up formations and attacking strategies when the two-point shot is available. It appears there is ‘safe’ value in taking close- versus long-range shots, while there is better value in taking long- versus mid-range shots. The combination of the two shooting positions (i.e. Goal Shooter and Goal Attack) may therefore represent a key consideration during the two-point shot period. Teams may benefit from using a combination of a tall ‘holding’ post shooter (i.e. remaining close to goal) with someone who is more proficient from long-range, as a means to take advantage of shots from the two varying distances. This combination may have the added benefit of providing a tall rebounder, increasing the chance of second shot opportunities from long-range misses. Teams may also need to adapt their attacking strategies in how they move the ball around and into the shooting circle, and how their two shooters position within the circle to promote shot opportunities from both close- and long-range. These practical considerations are also likely relevant to teams from a defensive perspective – whereby changes to defensive positions in line-ups and defensive strategies will require consideration in response to their opponent.”*

**C1.5: The implementation of the two-point scoring rule is an interesting topic and has potential for numerous future research applications. On top of the already discussed practical considerations are, obtaining data about players behaviours both qualitative and quantitative, how does this influence international matches that have traditional scoring methods, how the shooting from long range changes as a function of match status etc.**

R1.5: We wholeheartedly agree that the new rules implemented (i.e. two-point shooting, rolling substitutions) provide a foundation for future research as to how changing fundamental aspects of the sport influence strategy, performance and player behaviour. We have included a brief point on this directing to its importance in future netball research in the revised submission (see lines 442-443 in revised submission).

*“Understanding the impact of the new two-point rule on team/player shooting behaviours and success is an important area for future investigations.”*

**C1.6: I believe the study would be better suited to a comparison of a SSN season with the old scoring rule and the new scoring rule. As it is, it is quite speculative, and could potentially have a negative influence on match tactics given the data does not represent the actual state-of -play.**

R1.6: We agree with reviewer #1’s general perspective here – in that a comparison of the current rule to existing rules is required. However, the focus of the present study (with our analysis conducted before the season) was to examine the potential impact of the rule – particularly given that teams were given only a few weeks notice prior to the season, with no further practice matches available. Therefore, we took an applied approach and considered how sports scientists who may have been involved with one of the teams could consider the data available to make informed evidence-based decisions. In such situations, reliance on ‘old’ data is necessary to understand what might happen – and combine this with coach/player understanding of the sport to predict the impact of the rule change. In a practical context, teams cannot wait for until they have current data to make decisions on what they will do when new rules like this are thrust upon them, hence we wanted to capture this approach and provide an understanding of how existing data could be applied in such a context. We (and likely others too) have every intention of assessing data from the current season with the rule change implemented – and hope that the present study can provide some foundational aspects for the approaches researchers can take and compare their own data to.

**Reviewer #2**

**C2.1: Thank you for the opportunity to review this paper which was very well presented and very topical within the Australian netball scene. Whilst this may seem like a narrow focus given the rule change is related to a domestic competition, the fact that Australia is one of the world’s leading netball nations suggests that rule changes such as this could also be incorporated in future international standard rules competitions as they are currently in the shorter version of Fast5 netball. This paper has used data from recent years to develop probabilities and possibilities associated with goal shooting and scoring under the new rules. Whilst there are many factors in a game that will influence the potential scoring strategies and thus probable outcomes as shown in this paper, it has provided a very good starting point. No doubt future studies will be able to assess actual strategies used and tactics developed in response to this rule change, however it is important in the early days to have some data to inform practice.**

R2.1: Thank you for this and other comments made in your review. We agree (and this was a central aim of our approach) that this original study will form a base for discussion and further analysis of new data from the current season (and potential future seasons) where the rule is in place. Reviewer #1 raised some similar concerns surrounding this notion, and hence we refer you to our above responses – specifically R1.1 and R1.6 – for further discussion on this point.

**C2.2: Line 66-67 - it is not clear why there might be a higher degree of difficulty compared to the traditional one point conversion**

R2.2: Thank you for pointing this out – our original statements here assumed knowledge of National Football League rules. We have amended this section of the manuscript to provide an explanation of the difference between one- and two-point conversions following touchdowns (see lines 62-69 in revised submission).

*“The National Football League (NFL) introduced the choice of a two-point conversion option following touchdowns in 1994 – an option to run an additional on-field play from the two-yard line to reach the end zone for two points versus the standard place kick from 15-yards (or from 2-yards prior to 2015). This allows teams to potentially increase the value of a touchdown, but at a likely higher degree of difficulty (i.e. success rate has typically fluctuated around 50% across years) compared to the traditional one-point conversion (i.e. success rate typically remains over 90% across years) option.”*

**C2.3: Lines 81-87 - interesting comparison to the introduction of the three point shot in basketball and the increasing use of three point shots in today’s game. The authors have suggested why the three point shot is more popular however these same reasons would have been applicable at all times. Is it possible to suggest why it has been more used since 2000?**

R2.3: This is an interesting point to consider – and there isn’t necessarily any evidence to support a reason for why this lag in uptake of a more focused three-point strategy exists. Our speculation is that an increased reliance on statistics and analytics in sport (i.e. the ‘Moneyball’ approach) has played a role in this. A brief discussion on this potential reasoning has been included in the revised submission (see lines 83-86 in revised submission). This new discussion point somewhat repeated information in the initial part of this paragraph from the original submission (*“While the three-point line was introduced over 40 years ago, recent developments in the use of analytics to inform decision-making in the sport has altered many teams approaches to scoring.”*) – and thus this sentence has been removed from the revised submission.

*“It is unknown why a lag in predominant uptake of the three-point shot exists from when the rule was introduced in 1979 to post 2000. However, we speculate that the increased reliance on statistics and analytics for informing decision-making in sport may be a factor.”*

**C2.4: Lines 91-99 - the authors state that the aim of the paper is to answer a series of questions regarding implementation and potential use of the two-point rule. I would suggest that question one and two are retained as research questions that can be addressed with the data provided, however, I would recommend that RQ3 and 4 are reworded and refined to reduce the subjectivity and more closely align to the data that is available i.e., remove “should” and “could”; RQ4 – make reference to the comparison between SSN and Fast5.**

R2.4: We agree with reviewer #2 here and have reworded the research questions to hopefully better align with the data used to answer these questions (see lines 99-104 in revised submission).

*“(iii) how much added value does the two-point rule offer, and theoretically what proportion of shots from the two-point ‘outer circle’ may maximise a team’s scoring opportunity during the five minute period where the shot is available”*

*“(iv) compare shooting success between Fast5 and Suncorp Super Netball to examine how the situational context of having a two-point shot available (i.e. as in Fast5) might impact shooting success”*

**C2.5: Line 113 - each match was coded using the relevant broadcast footage - this could be deleted as line 108 refers to the broadcast video footage.**

R2.5: The suggested amendment has been made in the revised submission (see line 118 in revised submission).

*“Each match was coded using the relevant footage.”*

**C2.6: Line 125 - please confirm whether it was the radial distance that was calculated.**

R2.6: The assumption from reviewer #2 here is correct – and this has been specified in the revised submission (see line 130 in revised submission).

*“The radial distance in the coded XY coordinate from the designated goal end was then multiplied by the coordinate unit (i.e. 0.1525m) to determine the distance the shot was taken from goal.”*

**C2.7: How many games were analysed from the Fast5 format?**

R2.7: Thirty-six Fast5 matches were analysed. This detail has been added to the revised manuscript (see line 133 in revised submission).

*“We also used shooting statistics from International Fast5 matches (n = 36) across the 2016 and 2017 Fast5 Netball World Series in this study.”*

**C2.8: Did the goals analysed include penalty shots?**

R2.8: Yes, this included all shots across play in matches – as this aligned with our approach across the Super Netball matches. This distinction has been made in the revised submission (see lines 143-144 in revised submission).

*“Specifically, we extracted the total number of made and missed shots (including those in regular play and penalty shots) for the one- and two-point areas across all matches from all teams.”*

**C2.9: Line 178 - suggest inclusion of reference to Suncorp Super Netball.**

R2.9: The suggestion has been included in the revised submission (see line 183 in revised submission).

*“First – we calculated the proportion of shots taken from the respective 0.5m distance bins across a typical five-minute period by averaging the total number of shots from these distances across the entire duration of the Suncorp Super Netball season (i.e. 56 matches x 60 minutes each).”*

**C2.10: The methodology used to answer questions three and four provides an interesting perspective.**

R2.10: Thank you for this comment. No amendments made.

**C2.11: Lines 223-227 – this is really interesting data and I would have found it easier if it was included in Table 1.**

R2.11: Great point! We have moved the proportion of shots in the different distance bins to Table 1 in the revised submission (see lines 220-222 in revised submission).

**C2.12: By the very nature of the data and the aim of the paper the discussion is very speculative but it does present a range of possible scenarios which demonstrates a good understanding of the game and these are acknowledged as being speculative as you cannot guess what will happen in the game.**

R2.12: We agree with reviewer #2 around parts of the discussion needing to be speculative considering this analysis was undertaken on past data and prior to the 2020 season commenced. As such we have tried to ensure this is understood at important parts of the manuscript, and is particularly acknowledged in the limitations. As noted earlier, reviewer #1 raised some concerns around this which we have responded to above (see R1.1 and R1.6).

**C2.13: Likewise the limitations and conclusions are appropriately stated.**

R2.13: Again, thank you for this comment. No amendments made.

**Editorial**

**CE.1: very long paragraphs or lack of throughout the paper - these could be reviewed.**

RE.1: Efforts have been made to break up paragraphs more thoroughly through the manuscript. New paragraphs have been inserted at line 50 of the introduction; and lines 294, 334, 343, 390, 403, 434, 445, 448 and 457 of the discussion; and line 471 of the conclusions.

**CE.2: Abstract Line 17 – though should be through.**

RE.2: The recommended change has been made in the revised submission (see line 17 in revised submission).

**Additional Changes**

- As the new overtime rules were altered after our submission, but prior to the Super Netball season starting, we have removed any mention of these from the revised submission (e.g. in abstract, introduction, Figure 1 caption and discussion).
- An appropriate apostrophe has been added in the tile (i.e. Netball’s New Two Point Rule) as this was a grammatical error in the original submission.
- As per the journal requirements, we have checked the style guidelines for title pages and manuscript body and can confirm that our submission meets these requirements. We can also confirm that the figure files associated with the submission meet the designated naming conventions.
- Since our original submission, the Suncorp Super Netball rules around bonus points for winning a quarter were removed. Our statement in the original submission – *“The Suncorp Super Netball league also awards an extra point on the competition ladder for each quarter a team wins – making any additional scoring within each quarter beneficial”* – has been removed from the revised submission to avoid confusion.
