# Supplementary material for: When does risk outweigh reward? Identifying potential scoring strategies with netball’s new two-point rule
Source: PLoS One. 2020 Nov 23;15(11):e0242716. doi: 10.1371/journal.pone.0242716 (PMC7682905; doi:10.1371/journal.pone.0242716)
Supplement: Attachment — Submitted filename: Response.docx [file pone.0242716.s002.docx]

**AUTHORS RESPONSE TO REVIEWER COMMENTS**

Comments from reviewers have been presented in **bold** **text**; and comments from reviewers #1 and #2, and editorial comments have been listed as C1.X, C2.X and E.X, respectively, with ascending numbers used to label these comments. Responses to comments are in plain text and have been labelled using a similar format (e.g. R1.1 indicates the response to comment C1.1). Where revisions have been made they are referred to using line numbers of the revised manuscript, and these changes have been highlighted in the revised manuscript using **bold red text**.

**Reviewer #1**

**The authors have done a good job responding to the comments. Well done. This is an interesting first step, hopefully the authors continue this line of research given that there is now a full season of data with the rule change.**

**C1.1: I do have one more comment that I think could strengthen the article, and have a broader message for sport in general. In the changes made in response to reviewer comment C1.4, there is an opportunity to highlight the complexity inherent within netball and team sports in general. For example, how one rule change will create multiple emergent properties that come about from it. For instance where you now talk about the influence of the new rule on tactics, substitutions, and strategy, through to recruitment etc, I think the message needs to be conveyed that changing one rule will have knock on systemic influences and that need to be considered. For example, I think about the 6, 6, 6 rule introduced to AFL and the negative unintended consequences that came about due to this rule change. Also, I remember the backlash from some high-profile netball coaches and players regarding the lack of consultation about the rule changes. Not all changes will have the intended effect, and consideration of negative consequences needs be front of mind for sport stakeholders.**

**The following reference can be used to support this argument about complexity of netball. Mclean S, Hulme A, Read, G, Mooney M, Bedford A, Salmon, P. (2019) A systems approach to performance analysis in women’s netball: Using Work Domain Analysis to model elite netball performance. Frontiers in Psychology. 10:201. DOI: 10.3389/fpsyg.2019.00201.**

R1.1: This is an interesting point, and one that certain requires further investigation now that we have seen a full season with the rule (and will soon see a second season) take place. We have included a brief paragraph in the revised manuscript touching on some of these potential ‘knock on’ effects or broader issues (see line 321-327 in marked/tracked manuscript). While only mentioning a couple of issues, we feel these are some of the key elements that are already presenting with this new rule – but also note that further research is necessary in order to truly understand these broader impacts.

*“The aforementioned tactical considerations are focused on the performance or defence of the two-point shot. However, netball can be viewed as a complex system with many integrating parts[11] – and one rule change may have a ‘knock-on’ effect across the sport. We highlight the need to consider shooting circle line-ups, yet teams may also require varied mid-court line-ups including players more effective at generating long-range shots from circle feeds. The introduction of a new scoring rule could also impact on the overall make-up, and how perceived value is distributed across a team. Player’s expressed concerns over the introduction of the two-point rule, mostly due to a lack of consultation, but also surrounding how a rule that potentially boosts the value of shooting positions could impact on the value of defensive players. If teams perceive a need to attract shooting players through high-value contracts, this may lower the league-wide value of defensive players and impact their earning potential through the sport. Player discontent, in general, may also lead to other various unintended negative consequences. Our study was focused on assessing performance strategies related to the new two-point rule, hence understanding these potential systemic effects are outside the scope of our work. The broader impact of such singular rule changes must, however, be considered in future research.”*

**Reviewer #2**

**Thank you for your considered responses which have added little extra detail to the paper. I think the rewording of the research questions is much better.**

**C2.1: With respect to my comment R2.3 I was referring more to the difference between the statistics reported from early 2000 until now, rather than the lag between 1979 to post 2000, and as such I don’t think this additional information in lines 84-86 is necessary as it is not clearly stated what the percentage was at 1979, so comparisons cannot be made. However it is stated that from early 2000’s to now, the make-up of the shots has changed from 35% mid-range shots and 18% three-point shots, to then flip to 15% mid-range and 35% three-points. My question was why has this flip occurred as the reasons for using the three-point shot as described by the authors would have also been relevant in the early 2000’s or in fact when it was first introduced …..so why now (or over the years) has this increased use of the three-point shot developed? It was a slow change maybe….so is that what we will expect in netball?**

**I am not sure anyone would have a definitive answer unless there has been a change in something else, but it is likely to be just an increased ability of players to shoot the 3 pointer, or a change in the acceptance of risk in taking these shots maybe? Unless the authors have a different response I would suggest removing the additional comment as stated above (lines 84-86), and I am happy to leave the content as it was originally.**

R2.1: This is a fair point to address – as the comment we added did not mesh with the statistics provided (i.e. numbers from 2000 onwards). This was a mistake on our behalf, and the comment we included (i.e. *‘…we speculate that the increased reliance on statistics and analytics for informing decision-making in sport may be a factor’*) is actually reflective of the shift from the year 2000 onwards. We believe this is an important hypothesis to include in the manuscript – as it highlights the potential impact of statistics and performance analysis analytics in influencing decision making in modern sport. As such, we have amended it to reflect the notion that this has potentially become more relevant in modern times (see line 85-88 in marked/tracked manuscript).

*“It is unknown why a lag in predominant uptake of the three-point shot exists from when the rule was introduced in 1979 to post 2000; nor is their direct evidence to support why teams have dramatically shifted away from mid-range to three-point shots in recent years. However, we speculate that the increased reliance on statistics and analytics for informing decision-making in sport may be a factor in this modern era shift.”*

**C2.2: Line 114 and 119 - I think there was a misunderstanding with my comment, this insertion is not necessary as it is clearly stated on line 114 that broadcast video footage was used and on lines 115 and 116 that this video footage was coded by a single individual. I would suggest the removal of this new sentence.**

R2.2: As suggested, the originally inserted sentence (*‘Each match was coded using the broadcast footage’*) has been removed from the revised manuscript (see line 119 in marked/tracked manuscript).
